# A qualitative study of the communication and information needs of people with learning disabilities and epilepsy with physicians, nurses and carers

**DOI:** 10.1186/s12883-018-1235-9

**Published:** 2019-01-19

**Authors:** Jerry Paul K. Ninnoni

**Affiliations:** School of Nursing and Midwifery, Department of Mental Health, University of Cape Coast, Cape Coast, Ghana

**Keywords:** Learning disabilities, Epilepsy, Communication, Carers, Physicians, Nurses

## Abstract

**Background:**

Living with a chronic condition such as epilepsy can have a debilitating effect on the individual and their carers. Managing epilepsy among people with learning disabilities may present a challenge because of limited communication and may require a multidisciplinary approach. The study investigated the communication and information needs of people with learning disabilities with epilepsy and their physicians, nurses and carers.

**Methods:**

Qualitative designed was adopted to collect data from 15 community-based people with mild learning disabilities with epilepsy and 13 carers. Recorded data were transcribed verbatim and analysed thematically.

**Results:**

A range of findings emerged related to patient communication and information needs. These included: Knowledge regarding epilepsy; involvement; honesty and openness when giving information and consistency in provision of information.

**Conclusion:**

People with learning disabilities with epilepsy and their carers would like to know more about epilepsy, to be more involved decision makings through communication in the management of epilepsy to enable them feedback information regarding their health.

## Background

Effective communication is more than just providing information but transactional between the communication partners. It involves understanding the emotion and reasons behind the information. In addition to being able to convey a message, there is a need to listen in a way that gains the full meaning of what is being communicated and the other party feels being heard and their views considered. Thus, effective communication is central to the management of service users who have learning disabilities with epilepsy and their doctors, nurses and carers.

Seizure control maybe one of the main goals for medical and nursing staff as well as for people with learning disabilities and their carers [1]. The individual with epilepsy will need to have knowledge of the condition and be able to communicate information regarding seizures, medications and side effects [2, 3]. It is claimed that people with learning disabilities are at greater risks of seizures compared with the epilepsy population overall [4–6] and mortality is increased among the learning disabilities population [7]. Also, people with epilepsy who live in institutional settings have better seizure control when compared with those in community settings [8–13] and this may reflect the availability of specialist services in residential settings. Following deinstitutionalisation, there are increased numbers of people with learning disabilities and epilepsy residing in communities either living independently or supported by carers who may have limited knowledge and information regarding epilepsy [14, 15]. However, patients and carers’ knowledge and information regarding epilepsy may facilitate communication with health professionals such as doctors and nurses.

Non-adherence may imply a refusal to seek healthcare, non-participation in health management or failure to follow doctors’ instructions [16, 17]. It may also take other forms for example, the information or education given by healthcare professionals is either misunderstood, administered wrongly or the information is ignored completely [18, 19]. All these could be due to a range of factors including communication lapses and inadequate information provision [20, 21]. The philosophy of concordance advocates a decision-making process where patients can feel more involved in communication and more comfortable with their treatment [22]. It is argued that adherence is a multivariate construct that is determined by the interplay of many factors [14, 20]. Aspects may reflect the complexities of treatment regimes, level of support and the individuals living circumstances [14, 23]. A study by Eastock and Baker [24] reported that failure to comply with treatment is common among people with epilepsy compared with other chronic conditions. And the reasons for non-adherence included; lack of understanding of why it was necessary to adhere to treatment and the level of information provision, doctor-patient relationship, psychosocial factors and patient characteristics [24, 25]. It has also been found that patients reporting with side-effects are more likely to be non-adherent [20, 25]. This may be due to poor communication and are reported higher among individuals with learning disabilities who are also more susceptibility to unidentified side effects [20, 26, 27].

Partnership has been identified as a key determinant of patient satisfaction and the involvement of the patient in decision-making is important to promoting adherence [28]. Studies have shown that patient satisfaction and adherence are ultimately linked to patient involvement in the treatment [18, 29]. Patients who feel their healthcare professionals communicate well with them and actively encourage them in their own care are more likely to adhere to treatment [30–33]. Service providers are required to develop a reciprocal relationship where the exchange of information, identification of problems and the development of solutions are shared with the patient where they can input in the communication exchanges [34, 35]. It is asserted that when patients see themselves as partners and as problem solvers it may motivate them to exchange information more freely and they are more likely to adhere to their treatment recommendations [34, 36]. Yet, it is unclear to what extent service users with learning disabilities and epilepsy are involved in communication regarding services provision. Also, people with learning disabilities are more likely to acquiesce during conversations [37, 38]. Therefore, there is a need to encourage and support people with learning disabilities to express their views and feelings [39–43]. Community-based people with learning disabilities and epilepsy may want to lead an independent life and adopt a more consumerist perspective regarding the services they receive and thus there is a need for effective communication.

However, little is known regarding the views of people with learning disabilities with epilepsy and carers communication with doctor and nurses. There is a paucity of research that investigated people with learning disabilities and epilepsy views regarding communication [44]. To a larger extent, communication studies regarding people with learning disabilities are more common among children populations [45–47], are based on institutional or residential settings and included people with severe to profound learning disabilities [48, 49]. However, community-based adults with learning disabilities and epilepsy may have different communication needs and expectations. In addition, studies involving people with learning disabilities are mostly one-sided regarding the individuals’ perspectives of communication. The individuals with learning disabilities views regarding communication are often not reported. Studies focused predominantly on staff views and developing their communication skills to the neglect of service user’s regarding communication. Thus, an important aspect is missing as communication is at least a two-way process. Besides, little is known regarding people with learning disabilities with epilepsy views and experiences regarding communication with doctors, nurses and carers. The study sought to bridge this gap by giving a voice to people with learning disabilities and epilepsy to express their views regarding communication with doctors and nurses.

## Methods

This study utilised a descriptive qualitative approach using face-face interviews to explore the views and experiences of people with learning disabilities and epilepsy and their carers regarding communication with physicians and nurses.

### Setting

The study was conducted in a rural area in the North of Scotland as part of The Managed Clinical Network for Epilepsy which comprises the Community Learning Disability Teams, Epilepsy Field-workers, Neurologists, General Practitioners and Consultants.

### Population and sampling procedure

Purposive sampling techniques were used to identify the people with epilepsy and learning disabilities who met the inclusion criteria. Participants were recruited through the learning disabilities teams who identified the participants within the locality. Inclusion criteria were adults with mild learning disabilities with confirmed diagnosis of epilepsy; receiving learning disabilities services as identified by the consultants and aged 16–50 years. Only people who gave written informed consent were sampled.

### Ethical approval

The study was approved by the Grampian Research Ethics Committee.

### Data collection

Data were collected using semi-structured interviews. Data saturation was reached with 15 services users and 13 carers. Each interview lasted about 30–40 min. Interviews were taped with the participant consent. Participants were interviewed either at the Surgery, Day centre or at home as deemed convenient by the participant. Two carers were excluded from the study (one care-worker did not meet the inclusion criteria and the other declined to participate in the study). All participants except two carers consented to the interviews to be taped. The researcher was permitted to keep written notes of the non-recorded interviews. Interview questions were largely about communication exchanges and information provision between services users, doctors, nurses and carers regarding epilepsy. Specific questions were on patients and carers’ information needs regarding epilepsy.

### Data analysis

Thematic data analysis approach [50] was adopted. Data were transcribed verbatim. Transcripts of about 50 pages were printed. The transcripts were read through several times to obtain a sense of the whole [51]. Participants experiences of communication and information provision with healthcare professionals and carers regarding epilepsy and related issues constituted the unit of analysis. Each interview was read, and margins and notes made to form initial codes [52]. The interview was divided into meaning units that were condensed such as communication exchanges, information provision, involvement, listening and understanding. Significant statements and commonalties were organised into themes representing clearly define response category [50]. Emergent themes were documented and discussed to ensure inter-rater reliability. In the event of disagreements regarding themes the transcripts were revisited and analysed until consensus was reached. Identified themes were matched with the initial category.

### Participants characteristics

Two carers had learning disabilities but not epilepsy and one carer also had epilepsy but not a learning disability. In terms of demographic characteristics 61% of the participants were females [22] and 39% [12] males (Tables 1 and 2 respectively).

**Table 1:** Characteristics of patients

Patients (Codes)	Gender	Place of interviews	Relationship with carer
PA	F	Daycentre	Patient/daughter
PC	F	Daycentre	Patient
PE	M	Home	Patient/husband
PG	M	GP Surgery	Patient
PI	M	Home	Patient
PK	F	Home	Patient
PM	M	Home	Patient
PN	F	Home	Patient
PP	M	Home	Patient
PR	F	Home	Patient/wife
PT	F	Home	Patient
PV	F	Home	Patient
PX	F	Home	Patient
PY	M	Home	Patient/husband
PAa	F	Home	Patient

**Table 2:** Characteristics of carers/care-workers

Carer code	Gender	Interview location	Relationship with patient/s
PB	F	Daycentre	Family carer
PD	M	Daycentre	Care-worker
PF	F	Home	Family carer
PH	M	GP Surgery	Care-worker
PJ	F	Patient home	Care-worker
PL	M	Patient home	Care-worker
PO	F	Patient home	Care-worker
PQ	F	Patient home	Care-worker
PS	M	Patient home	Family carer
PU	M	Patient home	Care worker
PW	F	Patient home	Care worker
PZ	F	Patient home	Family carer
PAb	F	Patient home	Care worker

Overall, the nature of seizure control in most of the patients was reported as good by patients and carers. However, in a few participants, the epilepsy control was reported as poor. Participants were given the choice to decide the location for the interview for example, at their own home, day centre, a GP surgery or at the university. The majority 79% [21] preferred and were interviewed at home and the others 21% [7] at either the GP Surgery or Day-care centres (Tables 1 and 2). Regarding patient/carer relationships, 11 were care-workers with only four family carers. The types of support provided for patients was mainly based on activities of daily living with very little or no involvement in health managements.

## Results

The study yielded a range of concerns relating to patients (service users) and carers’ communications needs with doctors and nurses. Concerns expressed by both service users and carers related to knowledge and the inadequacy of information provision by doctors and nurses. However, this appeared to vary among different professional groups. Service users reported they would like to be more involved in decision making regarding their care. They wanted interactions and relationship with doctors and nurses built on trust and information provision to be consistently shared. Four main themes characterised patients and carers’ communication and information need with doctors and nurses.

### Theme 1. Knowledge regarding epilepsy

Service users and carers expressed the desire to communicate effectively but appeared to be constrained by their limited knowledge regarding epilepsy, seizures and medication. They both expressed the need to know more about epilepsy and would value some form of training to improve their knowledge on epilepsy and seizures:

*‘……a gap in knowledge because I don’t know obviously enough about as much as I should because I work with her and more knowledge for what to look out for, just general knowledge of epilepsy would be ideal’ Care worker PAb*

*‘I understand there are training programmes, but I have not been yet’ Care worker PAb*

*‘She [carer] would like to know more about it as I would like. That is what she needs to do, to learn something about epileptic fits’ Patient PE*

*‘I asked, and I was given a DVD video for a shot and return it but it does not teach me anything. I would like to know more about epilepsy of what to do if he takes a ‘turn’ [seizures]. What I need is somebody to come up and sit down with me and tell me more about epilepsy is all I need but everybody is busy and I am not the pushy type of person’ Family carer PF*

### Theme 2. Involvement

Overall, service users and carers’ communication purpose and needs with doctors and nurses were based on a wide range of things. However, involvement in decision making was central to their communication needs, they want to be more involved in communication. Service users have consistently expressed concerns regarding their lack of involvement in communication although this appears to vary among doctors and nurses:

*‘The doctors will tell you, take your tablets and that is it, whereas people like* [nurse] *will help you, talk to you about it, sit down face-to-face whereas doctors will want you out of the door’ Patient PP*

Similarly, carers expressed their wishes to be more involved in decision-making regarding patients and to be able to advocate for patients when necessary but felt they have often been side-lined by the doctors when making decisions for service users.

*‘I would just like to be able to talk a lot more about her so that if I am worried, I can discuss it with them and between us we can put it right hopefully’ Family carer PB*

*‘………I tried to change her appointment because she had no time to do it or something and I phoned the reception and say could she change it from this time to that and they said they are sorry they could not do it because it was confidential and that was just stupid because it has nothing to do with medicine. She was just asking to change but I suppose maybe they do get some ‘nutters’ who would do it for fun but I am her mum. Is stupid because they knew I am her mum they can identify the two of us together. I think if you know the way she is they should be a bit more forthcoming because she can’t always relay it to me you know’ Family carer PB*

### Theme 3. Honesty and openness in the provision of information

Other concerns that were repeatedly expressed by service users were related to the issue of withholding or concealing information. Service users wanted more discussions regarding changes to medications to be openly and honestly discussed with them. It appears there is conflict between autonomy and paternalism as reported:

*‘I find it very difficult sometimes you have a very bad back problem. I used to get pain killers for bad back ache, but they have taken them out these pills that I used to, they don’t have any side effects with my medications but they have taken them off’ Patient PT*

‘*There was one doctor I think last week I have been on medication for my depression and one doctor tries to reduce it because I have not been getting the truth that ok...* [doctors] *have reduced it and all that my antidepressant but on Friday I have to go past and get it put back to normal’ Patient PC*

### Theme 4. Consistency in the provision of information

Furthermore, other concerns related to conflicting information from doctors and nurses and the need for consistency in the provision of information. Service users and carers would value consistency in making decision where both carers and services are aware of decisions that are taken:

*‘They have put a stop to my other tablets that I used to take but I don’t know why, I think the….* [nurse] *said the learning disabilities team agreed on that but when I spoke with my Mum, my Mum said I was best taking two at lunch time instead of the one’ Patient PT*

This view was also shared by carers’ who advocated for their involvements in multidisciplinary team meetings to ensure that information is consistently shared:

*‘There is a stumbling block because is happening right now, she is coming to me with her problems but she not sharing the same problems with her GP, she not sharing the same problem with the Psychiatric nurse, but I feel uncomfortable going to the GP because I will be crossing boundaries here and I rely on her to convey appropriate information’ Care worker PD*

*‘But sometimes I just wish that there was a bit more communication with the support workers from the doctors and nurses but then everybody is busy’ Care worker PQ*

Persistent concerns expressed by both service users and carers related to lack of trust. Service users expressed concerns regarding medication errors. This led to patients questioning whether they were getting the prescribed or wrong medication. This was reported to be affecting their trusting relationships with doctors and nurses:

‘*Medication is funny, there are so many things going on with my medication. For instance, the doctors got mixed up between my medication and my brother’s medications, how is that? I try to understand whether I take different, medications’ Patient PP*

*‘I have been to the hospital here for once I don’t know when, when I was four years, but they did not give me the right medicine. This is what I cannot understand they have all your notes there and everything but give you the wrong medicine sometimes’ Patient PM*

This view regarding errors in medication was corroborated by the carer’s own observation of the error in the medication:

*‘He [patient] asked me a few months ago to double check his tablets because he felt the tablets were wrong and I did, and the tablets were definitely wrong and we both went down to the doctors to get everything sorted out’ Care worker PQ*

Service users also reported that they are not being trusted by doctors especially when reporting their experiences with medications and side effects:

*‘I am finding that I am sweating a lot because of the dizziness, I get angry because trying to explain to the doctors, sometimes when you are telling them you wonder if they believe you, it makes me cross sometimes. But is actually, you are going through it and they are just sitting on the chair and you wonder if they are taking it all in’ Patient PP*

## Discussion

The study explored service users’ and carers’ views and experiences of communication with doctors and nurses. The findings of this study demonstrated the complexities of communication involving people with learning disabilities and epilepsy. The findings indicated that service users have numerous communications need with their doctors, nurses and carers which appears to be impacting on quality of life.

### Epilepsy knowledge and information

It emerged from the study that both service users and carers considered knowledge regarding epilepsy and medication as a significant tool that may enhance communication, but this appears to be limited and evident by carers’ lack of knowledge regarding epilepsy and seizures. Carers need for knowledge and information regarding epilepsy concurs with earlier studies in the general population [15] but has not been reported by people with learning disabilities and epilepsy. Kendall and colleagues [53] also reported similar findings in their study, within an epilepsy organisation, regarding carers’ information needs relating to medications and side effects. The Scottish Intercollegiate Guidelines Network (SIGN) guidelines for epilepsy recommend that adults and their carers have the right to accurate information about the condition including the specific epilepsy syndrome, its treatment and implications for everyday life [54, 55].

### Service users and carers involvement

Lack of involvement in issues relating to epilepsy with doctors and nurses was reported as a concern by service users. The study demonstrated that service users appear to value more egalitarian relationships with doctors and nurses and would like to input on issues regarding their health [38]. It is claimed that most healthcare policies fit under the banner of patient and public involvement where interactions between patients and health professionals are encouraged [56]. Therefore, improving communication between patients and professionals and a shift away from paternalism to a more patient-focused approach has the potential to improve patient care [56]. People with learning disabilities in the community may have different communication and support needs compared with institutional settings and may want to be more involved in decision making that reflect their living conditions. This was reflected in the current study however a follow up study with doctors may present a full picture of this finding.

### Trust and credibility

Service users reported a need for information to be honestly and openly discussed with them. However, they perceived the information they get from doctors and nurses as insufficient and often they contribute little to the discussions. This however, was reported to vary among doctors and nurses. It is argued that whilst health professionals may be primarily interested in symptom reduction [57], service users in this study want to have an open discussion with their health care professionals regarding the management of their conditions where they can input regarding their health. The findings also point to the perceived lack of honesty between service users and doctors and nurses [58, 59]. This related to the apparent concealing and withholding of information from service users. It is claimed that health professionals commonly withhold health information from patients with their tacit consent [60, 61]. It is further argued that withholding information from patients may be ethically permissible and more generally, that honesty is not always the best policy [61]. However, in contemporary health care practice, patients are increasingly expected not only to know their diagnosis but also have detailed information regarding treatment options and prognosis [60, 61]. Service users highlighted a lack of trust and credibility when communicating with doctors relating to medication and side effects. A service user reported a lack of trust with the doctors as negatively impacting on communication. Patients expressed concerns regarding the medication they receive, fearing that they may be receiving the wrong medication: The prevalence of medication errors had been reported in the general population [62, 63]. It is claimed that medication prescribing deficiencies are the most common cause of actual and potential adverse drug events [64–66].

Furthermore, service users have concerns relating to lack of trust with doctors especially regarding medication side effects. They reported that doctors do not appear to believe them when reporting side effects. This finding reflect previous studies in the general population which revealed misunderstanding and disagreements regarding medication side effects [19]. To a significant extent, doctor-patient relationship is dependent on trust; and effective communication is nurtured in trusting relationships [67, 68]. All health professional bodies, for example, the General Medical Council and the Nursing and Midwifery Council both emphasize the need to maintain trust with patients as a top priority. People with learning disabilities and epilepsy are more likely to be taking multiple medication and may be particularly susceptible to antiepileptic drug side effects [69–71].

The significance of trust between patients and health care professionals has been widely reported in the literature [72–74]. Trust is said to be vital to patient-doctor relationships and with other health care professionals and can mediate important behaviours and health outcomes [74, 75]. Patients perceive more trust in health care professionals who use more patient-centred in communication [74]. Other studies reported trust to be positively associated with the doctors’ experiences and also to be dependent on the patient-doctor relationships [73].

### Information sharing

This study showed that both service users and carers’ communication with doctors and nurses also related to the need to share information consistently regarding epilepsy. People with learning disabilities and epilepsy may have different communication needs that require multidisciplinary team approach and they need to have information consistently shared with carers, doctor and nurses. Providing accurate and consistent information about epilepsy and seizure managements are core tenets of patient-centred care [76, 77]. The importance of multidisciplinary approach to addressing the communication needs of adults with learning disabilities have been captured in the literature [78]. A similar study also reported that patients are confused by the conflicting advice from doctors and nurses [19]. It can be argued that people with learning disabilities are particularly vulnerable to medication errors due to possible communication difficulties and cognitive impairments. Therefore, consistent information sharing with nurses and carers in medication management may be crucially important.

### Limitations of the study

This is a qualitative study in a small geographical location in Scotland and thus no attempts are made to generalise the findings to a wider context. Also, study only focused on the views of service users and their carers but not doctors and nurses.

## Conclusions

Service users and carers communication experiences with doctors and nurses include: limited knowledge of epilepsy involvement in decision-making; maintaining trusting relationships with doctors and nurses and a multidisciplinary approach to health management involving carers to ensure information is comprehensive and consistently shared.

The findings reveal gaps in communication between doctors, nurses and carers. These may have implication for practice regarding the role of effective communication to improving epilepsy care among this population group. Also, doctors and nurses may need to adopt the domains of patient-centred models where the patients are more involved, and their views and beliefs respected in the encounter. The findings also implied healthcare professionals may need to reflect, adjust and adopt a more patient-centred approach when engaging with patients. Also, the findings suggest that policies focusing on the involvement of people learning disabilities are translated in clinical practice. The findings also have implications on education. Professional practice. Public awareness and health literacy must be increased, beginning with education of students’ nurses and other health professionals to improved doctor-patient communication skills.

## Abbreviations

PCC: Patient-Centred Care
SIGN: Scottish Intercollegiate Guidelines Network
